# Investigating rare pathogenic/likely pathogenic exonic variation in bipolar disorder

**DOI:** 10.1038/s41380-020-01006-9

**Published:** 2021-01-22

**Authors:** Xiaoming Jia, Fernando S. Goes, Adam E. Locke, Duncan Palmer, Weiqing Wang, Sarah Cohen-Woods, Giulio Genovese, Anne U. Jackson, Chen Jiang, Mark Kvale, Niamh Mullins, Hoang Nguyen, Mehdi Pirooznia, Margarita Rivera, Douglas M. Ruderfer, Ling Shen, Khanh Thai, Matthew Zawistowski, Yongwen Zhuang, Gonçalo Abecasis, Huda Akil, Sarah Bergen, Margit Burmeister, Sinéad Chapman, Melissa DelaBastide, Anders Juréus, Hyun Min Kang, Pui-Yan Kwok, Jun Z. Li, Shawn E. Levy, Eric T. Monson, Jennifer Moran, Janet Sobell, Stanley Watson, Virginia Willour, Sebastian Zöllner, Rolf Adolfsson, Douglas Blackwood, Michael Boehnke, Gerome Breen, Aiden Corvin, Nick Craddock, Arianna DiFlorio, Christina M. Hultman, Mikael Landen, Cathryn Lewis, Steven A. McCarroll, W. Richard McCombie, Peter McGuffin, Andrew McIntosh, Andrew McQuillin, Derek Morris, Richard M. Myers, Michael O’Donovan, Roel Ophoff, Marco Boks, Rene Kahn, Willem Ouwehand, Michael Owen, Carlos Pato, Michele Pato, Danielle Posthuma, James B. Potash, Andreas Reif, Pamela Sklar, Jordan Smoller, Patrick F. Sullivan, John Vincent, James Walters, Benjamin Neale, Shaun Purcell, Neil Risch, Catherine Schaefer, Eli A. Stahl, Peter P. Zandi, Laura J. Scott

**Affiliations:** 1 Weill Institute for Neurosciences, University of California San Francisco, San Francisco, CA 94158 USA; 2 Department of Psychiatry and Behavioral Sciences, Johns Hopkins School of Medicine, Baltimore, MD 21287 USA; 3 Division of Genomics & Bioinformatics, Department of Medicine and McDonnell Genome Institute, Washington University School of Medicine, St. Louis, MO 63108 USA; 4 Stanley Center for Psychiatric Research, Broad Institute of MIT and Harvard, Cambridge, MA 02142 USA; 5 Division of Psychiatric Genomics, Icahn School of Medicine at Mount Sinai, New York, NY 10029 USA; 6 Discipline of Psychology and Flinders Centre for Innovation in Cancer, Flinders University, Adelaide, SA Australia; 7 Medical Research Council Social Genetic and Developmental Psychiatry Centre, Institute of Psychiatry, Psychology and Neuroscience, King’s College London, London, UK; 8 Department of Biostatistics and Center for Statistical Genetics, University of Michigan, Ann Arbor, MI 48109 USA; 9 Division of Research, Kaiser Permanente Northern California, Oakland, CA 94611 USA; 10 Institute for Human Genetics, University of California San Francisco, San Francisco, CA 94143 USA; 11 Pamela Sklar Division of Psychiatric Genomics, Department of Genetics and Genomic Science, Icahn School of Medicine at Mount Sinai, New York, NY 10029 USA; 12 Bioinformatics and Computational Core, National Heart, Lung, and Blood Institute, Bethesda, MD 20892 USA; 13 Department of Biochemistry and Molecular Biology II, Institute of Neurosciences, Center for Biomedical Research, University of Granada, Granada, Spain; 14 Departments of Medicine, Psychiatry, and Biomedical Informatics, Vanderbilt University Medical Center, Nashville, TN 37232 USA; 15 Molecular & Behavioral Neuroscience Institute, University of Michigan, Ann Arbor, MI 48109 USA; 16 Department of Medical Epidemiology and Biostatistics, Karolinska Institutet, Stockholm, Sweden; 17 Department of Computational Medicine & Bioinformatics, University of Michigan, Ann Arbor, MI 48109 USA; 18 Department of Human Genetics, University of Michigan, Ann Arbor, MI 48109 USA; 19 Department of Psychiatry, University of Michigan, Ann Arbor, MI 48109 USA; 20 Division of Research, Cold Spring Harbor Laboratory, Cold Spring, Harbor, NY 11797 USA; 21 HudsonAlpha Institute for Biotechnology, Huntsville, AL 35806 USA; 22 Department of Psychiatry, University of Iowa Carver College of Medicine, Iowa City, IA 52242 USA; 23 Department of Psychiatry, Massachusetts General Hospital, Boston, MA 02114 USA; 24 Department of Psychiatry and Behavioral Sciences, University of Southern California, Los Angeles, CA 90033 USA; 25 Departments of Clinical Sciences and Psychiatry, Umea University, Umea, Sweden; 26 Division of Psychiatry, University of Edinburgh, Edinburgh, UK; 27 NIHR BRC for Mental Health, King’s College London, London, UK; 28 Department of Psychiatry and Trinity Translational Medicine Institute, Trinity College Dublin, Dublin, Ireland; 29 Medical Research Council Centre for Neuropsychiatric Genetics and Genomics, Division of Psychological Medicine and Clinical Neurosciences, Cardiff University School of Medicine, Cardiff, UK; 30 Institute of Neuroscience and Physiology, University of Gothenburg, Gothenburg, Sweden; 31 Department of Medical & Molecular Genetics, King’s College London, London, UK; 32 Department of Genetics, Harvard Medical School, Boston, MA 02115 USA; 33 Centre for Cognitive Ageing and Cognitive Epidemiology, University of Edinburgh, Edinburgh, UK; 34 Division of Psychiatry, University College London, London, UK; 35 Discipline of Biochemistry, Neuroimaging and Cognitive Genomics (NICOG) Centre, National University of Ireland Galway, Galway, Ireland; 36 Center for Neurobehavioral Genetics, University of California Los Angeles, Los Angeles, CA 90095 USA; 37 Department of Psychiatry, UMC Utrecht Brain Center Rudolf Magnus, Utrecht, the Netherlands; 38 Department of Psychiatry, Icahn School of Medicine at Mount Sinai, New York, NY 10029 USA; 39 Department of Haematology, School of Clinical Medicine, University of Cambridge, Cambridge, UK; 40 SUNY Downstate Medical Center, Brooklyn, NY 11203 USA; 41 Department of Psychiatry, SUNY Downstate Medical Center, Brooklyn, NY 11203 USA; 42 Department of Complex Trait Genetics, Center for Neurogenomics and Cognitive Research, Amsterdam Neuroscience, Vrije Universiteit Amsterdam, Amsterdam, the Netherlands; 43 Department of Clinical Genetics, Amsterdam Neuroscience, Vrije Universiteit Medical Center, Amsterdam, the Netherlands; 44 Department of Psychiatry, Psychosomatic Medicine and Psychotherapy, University Hospital Frankfurt, Frankfurt am Main, Germany; 45 Department of Psychiatric and Neurodevelopmental Genetics Unit, Massachusetts General Hospital, Boston, MA 02114 USA; 46 Departments of Genetics and Psychiatry, University of North Carolina, Chapel Hill, NC USA; 47 Molecular Neuropsychiatry and Development Laboratory, Campbell Family Mental Health Research Institute, Center for Addiction & Mental Health, Toronto, ON Canada; 48 Department of Psychiatry and Institute of Medical Sciences, University of Toronto, Toronto, ON Canada; 49 Analytical and Translational Genetics Unit, Department of Medicine, Massachusetts General Hospital, Boston, MA 02114 USA; 50 Division of Sleep and Circadian Disorders, Brigham and Women’s Hospital and Harvard Medical School, Boston, MA 02115 USA; 51 Program in Medical and Population Genetics, Broad Institute of MIT and Harvard, Cambridge, MA 02142 USA

**Keywords:** Genetics, Bipolar disorder

## Abstract

Bipolar disorder (BD) is a serious mental illness with substantial common variant heritability. However, the role of rare coding variation in BD is not well established. We examined the protein-coding (exonic) sequences of 3,987 unrelated individuals with BD and 5,322 controls of predominantly European ancestry across four cohorts from the Bipolar Sequencing Consortium (BSC). We assessed the burden of rare, protein-altering, single nucleotide variants classified as pathogenic or likely pathogenic (P-LP) both exome-wide and within several groups of genes with phenotypic or biologic plausibility in BD. While we observed an increased burden of rare coding P-LP variants within 165 genes identified as BD GWAS regions in 3,987 BD cases (meta-analysis OR = 1.9, 95% CI = 1.3–2.8, one-sided *p* = 6.0 × 10^−4^), this enrichment did not replicate in an additional 9,929 BD cases and 14,018 controls (OR = 0.9, one-side *p* = 0.70). Although BD shares common variant heritability with schizophrenia, in the BSC sample we did not observe a significant enrichment of P-LP variants in SCZ GWAS genes, in two classes of neuronal synaptic genes (RBFOX2 and FMRP) associated with SCZ or in loss-of-function intolerant genes. In this study, the largest analysis of exonic variation in BD, individuals with BD do not carry a replicable enrichment of rare P-LP variants across the exome or in any of several groups of genes with biologic plausibility. Moreover, despite a strong shared susceptibility between BD and SCZ through common genetic variation, we do not observe an association between BD risk and rare P-LP coding variants in genes known to modulate risk for SCZ.

## Introduction

Bipolar disorder (BD) is a serious mental illness affecting more than 48 million adults worldwide with a lifetime prevalence of 1–3% [1, 2]. BD is characterized by extreme episodes of mood elevation and depression, along with disturbances in thinking and behavior that often result in significant disability [3]. The high lifetime morbidity of BD and frequent suboptimal outcomes with available treatments present an urgent need to better understand the pathogenesis of BD. Prior studies have suggested a role for dysregulated neuronal signaling and synaptic plasticity [4–7], as well as pathways that regulate neurotransmitter function, neuronal development, and oxidative stress [8, 9]. However, a comprehensive characterization of biologic mechanisms in BD remains elusive. A better understanding is critical for development of effective therapies and personalized care.

BD is highly heritable (59–93%) within families, and monozygotic twins show higher concordance rates (40–80%) than dizygotic twins (5–30%) [10–14]. Genome-wide association studies (GWAS) have identified over 30 loci that contribute to BD susceptibility, implicating genes that encode ion channels, neurotransmitter transporters, and synaptic components [15–18]. Genes implicated by GWAS might also harbor rare variants [19–21], however this has not been studied in BD. Whole-exome sequencing (WES) in BD families has examined the role of rare exonic variation influencing BD risk. [22–26]. However, these studies have been small (discovery cohort <200 BD cases) [22–25], are single-cohort studies [22, 25], and only provide modest evidence implicating rare coding variants [23, 24, 26]. Overall, it remains unclear whether rare genetic variation significantly influences risk of BD.

BD shares clinical features and genetic susceptibility with schizophrenia (SCZ) [12, 18, 27–29], particularly through common genetic variation. In addition to the observation that BD and SCZ share a high genetic correlation due to common variation [30], the SCZ polygenic risk score (PRS) is associated with psychosis in BD, and the BD PRS is associated with manic behavior in schizophrenia [28, 31]. Studies of rare variation show that individuals with SCZ carry a higher burden of ultra-rare protein-altering variants across the exome, particularly in genes that are evolutionarily conserved, in genes that affect neuronal synaptic function, and that have been implicated through studies of de novo variation in SCZ and autism spectrum disorder [32–36]. Rare variants in these gene classes have not been systematically examined in BD. Despite the shared susceptibility between BD and SCZ through common genetic variation, it remains unknown whether rare variants in genes implicated in SCZ contribute to BD.

This study aims to test whether rare, functional protein-altering variation impacts susceptibility to BD. To this end, we examined the protein-coding sequences (exomes) of 3,987 individuals with BD and 5,322 healthy controls of predominantly European ancestry recruited across four studies to address the following questions: do individuals with BD carry a greater burden of rare functional coding variants (1) in their exomes overall; (2) in genes implicated by common variant GWAS in BD; and (3) in genes implicated through common and rare variant studies in SCZ, including neuronal synaptic and loss-of-function intolerant genes. We subsequently attempted replication of positive findings in an additional 9,929 individuals with BD and 14,018 matched controls.

## Methods

### Cohorts

The study protocol for the combined analysis was approved by the Johns Hopkins University Institutional Review Board. All human studies were approved by each respective institutional ethics review committee, and all participants provided written informed consent. We examined the exomes of 3,987 individuals with BD (3,615 with bipolar 1 disorder (B1D), 252 with bipolar 2 disorder (B2D), 91 with schizoaffective disorder bipolar type (SAB)) and 5,322 unaffected individuals from four case-control cohorts of predominantly European ancestry that comprise the Bipolar Sequencing Consortium (BSC) (Table 1, Figs. S1, S2, and Supplemental Methods). These included 1,712 cases and 1,844 controls from the Bipolar Research in Deep Genome and Epigenome Sequencing (BRIDGES) study [37], 961 cases and 1,039 controls from the Rare Bipolar Loci Identification Through Synaptome Sequencing (RareBLISS) study (NIMH Genetics Initiative repository) [26], 831 cases and 1,956 controls from Sweden (Swedish National Hospital Discharge Register and population-based registries), and 483 cases and 483 matched controls from the Kaiser Permanente Northern California (KPNC) cohort (Multi-ethnic Genome-wide Study of Bipolar1 Disorder and Resource for Genetic Epidemiology Research in Adult Health and Aging (GERA) cohort [38]). All studies were comprised of individuals of European ancestry except for KPNC, which included individuals of European (EUR, 192 cases, 192 controls), African American (AFR, 96 cases, 95 controls), Latino (LAT, 98 cases, 100 controls), and East Asian (EAS, 97 cases, 96 controls) ancestry.

**Table 1:** Bipolar disorder sequencing cohorts.

Study	Ethnicity	Total *N*	Case/Control *N*	Female (%)	B1D/B2D/SAB/NOS/UNK (%)	P-LP SNVs	Exonic SNVs	Exonic coverage: median (25–75%)
BRIDGES^a^	US-Caucasian	3,556	1712/1844	32.4	100/0/0	4,812	680,910	8 (6–11)
RareBLISS	US-Caucasian	2,000	961/1039	53.0	90.8/0/9.2	3,394	465,504	78 (42–101)
Sweden	Swedish-Caucasian	2,787	831/1956	53.8	65.8/30.3/0.4/1.7/1.8	1,945	328,066	28 (24–37)
KPNC-EUR	US-Caucasian	384	192/192	71.6	100/0/0	511	108,307	34 (22–51)
KPNC-AFR	US-African American	191	96/95	71.2	100/0/0	308	132,872	39 (25–57)
KPNC-LAT	US-Latino	198	98/100	72.7	100/0/0	298	101,368	40 (26–60)
KPNC-EAS	US-East Asian	193	97/96	63.7	100/0/0	334	88,884	39 (26–58)
All		9,309	3987/5322	47.1	90.6/6.3/2.3 0.4/0.4	9,883	1,328,324	

We examined protein-coding (exome) sequence data in 9309 individuals of predominantly European ancestry from four independent studies. Cases and controls were recruited at ~1:1 ratio except in the Sweden cohort (~1:2 cases to controls). The Kaiser Permanente Northern California (KPNC) cohort was comprised of four race/ethnicity groups: 40% non-Hispanic white, 20% African American, 20% Latino, and 20% East Asian. The number of unique exon variants (including exonic, splicing, and 3’ and 5’ UTR regions) is listed for each cohort and across studies.

*SNV* single nucleotide variant, *WES* whole-exome sequencing, *BRIDGES* Bipolar Research in Deep Genome and Epigenome Sequencing Study, *RareBLISS* Rare Bipolar Loci Identification through Synaptome Sequencing, *EUR* European ancestry, *AFR* African American ancestry, *LAT* Latino ancestry, *EAS* East Asian ancestry, *B1D* bipolar 1 disorder, *B2D* bipolar 2 disorder, *SAB* Schizoaffective disorder, bipolar type, *NOS* No otherwise specified, *Miss* missing specific diagnosis.

^a^Whole-genome sequencing was performed in BRIDGES. Exome sequencing was performed in all other BSC cohorts.

### Sequencing and quality control

Whole-genome sequencing (WGS) was performed in the BRIDGES cohort using the Illumina HiSeq 2500 system. Library preparation was performed using Nimblegen SeqCap EZ Exome (RareBLISS and KPNC) or Agilent SureSelect Human All Exon v2 kit (Sweden), and whole-exome sequencing (WES) was performed using Illumina HiSeq 2000 or 2500 systems in RareBLISS, Sweden, and KPNC. Paired sequence reads were aligned to the human reference build hg19 using BWA [39]. Variant calling was performed using the GotCloud sequence analysis pipeline [40] for WGS data in BRIDGES, and joint variant calling was performed using the Genome Analysis ToolKit (GATK) [41] for WES data within each of RareBLISS, Sweden, and KPNC. Genotypes with low sequence coverage or poor call quality were removed. Samples that were identified as population outliers, duplicates or relatives, or that failed study-specific sequencing metrics were removed. Variants with high missingness across samples, poor average genotyping quality, or found to significantly deviate from Hardy-Weinberg equilibrium were removed. A full description of sequencing and data quality control is provided in Supplemental Materials.

### Variant selection and annotation

Analysis was limited to single nucleotide variants (SNVs) that are bi-allelic and whose minor allele matched the alternative (non-reference) allele. Annotation of all variants identified from exome and genome sequencing was done using ANNOVAR, and pathogenicity was assigned according to 2015 American College of Medical Genetics (ACMG) criteria using InterVar, a computational implementation of expert panel recommendations for clinical interpretation of genetic variants (ACMG 2015 criteria) [42–44]. ACMG classification categories include pathogenic (P), likely pathogenic (LP), variant of uncertain significance, likely benign, and benign. Variants that were rare (maximum population-specific minor allele frequency [MAF] < 1% in the Genome Aggregation Database [gnomAD] [45, 46]), protein-altering (missense, splice site, stopgain, startloss, stoploss (none observed), and classified as pathogenic or likely pathogenic by InterVar were retained for analysis.

### Gene-level burden testing

To assess whether individuals with BD carry a greater burden of rare pathogenic or likely pathogenic (P-LP) variants in individual genes, we performed Firth logistic regression on the number of P-LP alleles within the gene per individual (adjusted for sex and at least five ancestry principal components) for each of seven groups (BRIDGES, RAREBLISS, Sweden, KPNC-EUR, KPNC-AFR, KPNC-LAT, KPNC-EAS), followed by a meta-analysis across groups using the weighted Z-score method. We calculated a one-sided *p* value for enrichment of P-PL variants in BD cases from the meta-analysis Z-score, and used a Holm-adjusted *p* value < 0.05 to report significant associations.

### Gene set selection

We assessed whether exomes of BD patients are enriched for rare functional protein-altering variants in genes implicated by the largest BD GWAS meta-analysis [18]. We defined the following three BD-related gene sets: 165 genes identified by physical proximity to variants in linkage disequilibrium (*r*^2^ > 0.1) with BD GWAS-associated variants (BD GWAS), 153 genes identified using the software package MAGMA (Multi-marker Analysis of GenoMic Annotation) [47] on BD GWAS data (BD MAGMA), and 81 genes identified through the intersection of the above two gene sets (BD MAGMA-GWAS). Given that BD shares genetic susceptibility with schizophrenia (SCZ) [12, 18, 27–29], particularly through common genetic variation, we examined genes implicated in a large schizophrenia GWAS (CLOZUK+PGC2) [48] by defining the following three gene sets: 623 genes identified by variants in linkage disequilibrium (*r*^2^ > 0.1) with schizophrenia GWAS-associated variants (SCZ GWAS), 550 genes identified using MAGMA on schizophrenia GWAS data (SCZ MAGMA), and 342 genes identified through the intersection of these two gene sets (SCZ MAGMA-GWAS). Given the strong impact of rare variant burden on SCZ susceptibility and the partial clinical overlap between BD and SCZ [34, 48–50], we also examined three gene sets previously implicated in SCZ rare variant risk: 3,055 synaptic genes whose mRNA are bound by the splicing factor RBFOX2 [32, 51] (Synaptic-RBFOX2), 1,033 synaptic genes whose mRNA are bound by FMRP [32, 52] (Synaptic-FMRP), and 3,230 genes classified as loss-of-function (LOF) intolerant from the large ExAC dataset [45] (LOF-intolerant).

### Gene set burden testing

We used two statistical methods to evaluate whether BD cases carry a higher burden of rare functional protein-altering variants (described above) than controls for each candidate gene set. First, we performed meta-analysis of Firth logistic regressions from each of seven groups described above. Second, we performed Cochran–Mantel–Haenszel (CMH) chi-square tests on the P-LP count in cases and controls across studies, and determined the empirical (one-sided) *p* value for enrichment of P-PL variants in BD cases by comparing this CMH chi-square statistic to a null distribution generated from a random selection of an equivalent number of genes in 10,000 simulations. We performed these random simulations to control for potential confounding due to variability in sample selection and sequencing methods between cases and controls within each study. We conducted all statistical analyses in R [53]. See Supplemental Methods for full burden testing methods.

### Replication

We examined exome sequence data in an independent cohort of 9,929 individuals with BD and 14,018 matched controls from the Bipolar Exomes (BipEx) collection (see Supplemental Materials). Of 3,639 BD cases with known subtypes, 2,684 were classified as B1D, and 955 were classified as B2D. After WES variant calling and quality control, we annotated exonic variants using InterVar, and extracted all rare (gnomAD maximum population MAF < 1%), protein-altering (missense, splice site, stopgain, startloss) SNVs that were classified as P or LP, as was done for the BSC studies. Statistical analysis was the same as used in the BSC analysis. Specifically, we examined the burden of rare P-LP variants in BD cases versus controls, and in B1D cases versus controls, within the three BD gene sets (BD GWAS, BD MAGMA, BD MAGMA-GWAS). We performed Firth logistic regression on the number of rare P-LP alleles per individual (adjusted for sex, 10 principal components, and the total burden of non-reference alleles) within each of six cohorts that comprised the BipEx replication sample (US, UK, Sweden-Umea, Sweden-Karolinska, Netherlands, and Germany), followed by a meta-analysis across cohorts using the weighted Z-score method.

### PRS analysis of common (GWAS) variants

Except for BRIDGES, which used genotypes from WGS, studies used previously published GWAS array data from European-descent individuals imputed into the 1000 Genomes or 1000 Genomes + Haplotype Reference Consortium reference panels to select variants for inclusion in polygenic risk score analysis [18, 38, 54–58]. We identified the set of variants present and well imputed (imputation *R*^2^ or info score >0.5) and with MAF > 0.01 in all studies and limited to the European ancestry sample in KPNC. We further filtered variants where any individual cohort frequency deviated from the mean frequency by >0.2, resulting in a final set of 2,855,373 SNPs for building polygenic scores.

To generate SNP sets and per SNP weights for building polygenic scores independent from the data in the current study, we started with association results from studies that participated in recent PGC2 analyses for bipolar disorder [18] and schizophrenia [59], then removed all studies with participants overlapping with BSC study samples and re-ran case-control association meta-analysis. These modified meta-analyses included roughly 11,000 cases and 17,000 controls for bipolar disorder and 19,000 cases and 28,000 controls for schizophrenia. In both the bipolar and schizophrenia association results, we extracted the subset of SNPs overlapping the high quality BSC variants described above and identified sets of variants to include in PRS analysis by clumping all SNPs based on LD (1MB windows and *r*^2^ > 0.1) using 1000 Genomes European individuals as the reference sample. For each trait, we selected variants at three different *p* value thresholds (*P* < 0.01, <0.05, and <0.1) to determine the effect of threshold on the strength of the PRS association with bipolar disorder. Each study then generated a PRS score for each individual based on genotype dosages and using log(OR) from the restricted PGC2 analyses as weights. Each group then tested for association using logistic regression of the PRS on bipolar case-control status in their BSC GWAS/WGS data adjusting for sex, genotype-based principal components, and where necessary, genotype batch. We combined results from all studies using inverse variance-weighed meta-analysis.

Code used in the analysis of the BSC samples is available at: http://metamoodics.org/bsc/consortium/bsc-case-control-workgroup

## Results

**Fig. 1:**
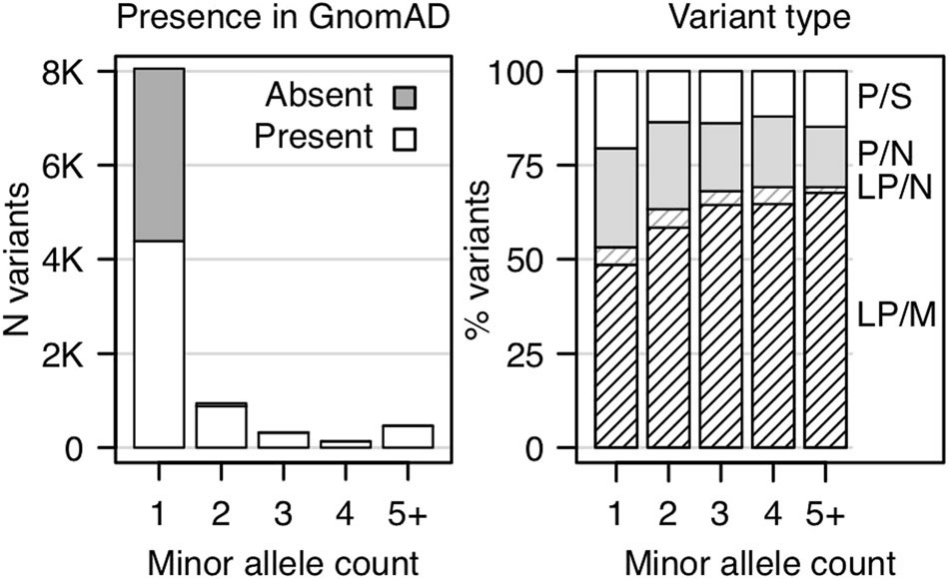
P-LP variant distribution. Minor allele counts for variants classified as P or LP from 9,309 individuals in the BSC cohort show that 81% of P-LP variants are singleton mutations. 38% of P-LP variants are not present in gnomAD. 44% of such variants are classified as P (predominantly splice site and nonsense variants), and 56% are classified as LP (predominantly missense variants). P pathogenic, LP likely pathogenic, S Splice site, N nonsense, M missense, ACMG American College of Medical Genetics, GnomAD Genome Aggregation Database.

Exome analysis in 3,987 individuals with BD (91% B1D, 6% B2D, and 2% SAB) and 5,322 unaffected individuals (Table 1, Table S1, Fig. S1) identified 1,328,324 exonic SNVs that passed quality control, of which 792,375 were rare (gnomAD maximum population MAF < 1%) and protein-altering (primarily missense, splice-site, and stop-gain); 9,883 (0.7%) of exonic SNVs were classified as pathogenic (P) or likely pathogenic (LP) using InterVar [42] (Table S2). Variants classified as pathogenic included 2,629 stop-gain and 1,963 splice-site altering SNVs affecting 2,076 genes. Variants classified as likely pathogenic included 5,152 missense, 463 stop-gain, and 1 stop-loss affecting 1,173 genes (Table S2). On average, each individual carried 0.72 pathogenic and 1.2 likely pathogenic SNVs across the exome. Most (81%) of P-LP variants were singleton mutations, and a significant proportion (38%) of such variants were not previously reported in the large gnomAD database (Fig. 1).

The exome-wide burden of rare synonymous variants did not differ between BD cases and controls (odds ratio [OR] = 1.00, 95% confidence interval [CI] = 0.9985–1.0004, *p* = 0.23), suggesting an absence of exome-wide bias in detecting rare variants across a heterogeneous group of sequencing studies. We subsequently restricted our analysis to rare protein-altering variants classified as pathogenic or likely pathogenic (P-LP) (Table S2). Examination of gene-level burden of rare P-LP variants did not reveal any genes that passed exome-wide significance (Table S3). Likewise, the exome-wide P-LP burden also did not differ between BD cases and controls (OR = 1.00, 95% CI = 0.97–1.03, *p* = 0.39), differing from sequencing studies of SCZ and developmental disorders of similar sample size showing an increased burden of rare coding variants across the exome [32, 42, 49].

**Fig. 2:**
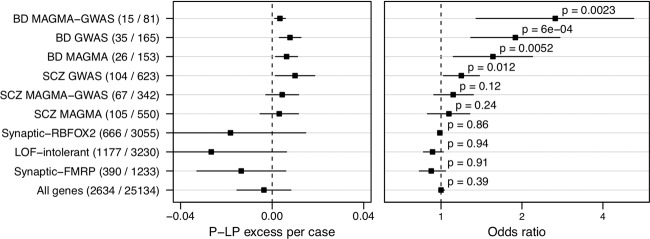
P-LP variant burden in candidate BD- and SCZ-related gene sets. Meta-analysis of Firth logistic regression of P-LP variants shows that BD cases in BSC cohorts appear to carry a higher burden of P-LP alleles in three BD GWAS-derived gene sets. Horizontal bars represent 95% confidence intervals. No enrichment of P-LP variants was observed in three schizophrenia GWAS-derived gene sets, in two neuron synaptic-related genesets (RBFOX2- and FMRP-related genes), in genes classified as LOF-intolerant, or in all genes across the exome. *P* values are one-sided for enrichment of P-PL variants in BD cases and derived from the meta-analysis Z-score. Numbers in parentheses represent the number of genes with P-LP variants and total number of genes within each gene set, respectively. BD bipolar disorder, OR odds ratio, P-LP pathogenic or likely pathogenic, GWAS genome-wide association study, LOF loss-of-function.

**Fig. 3:**
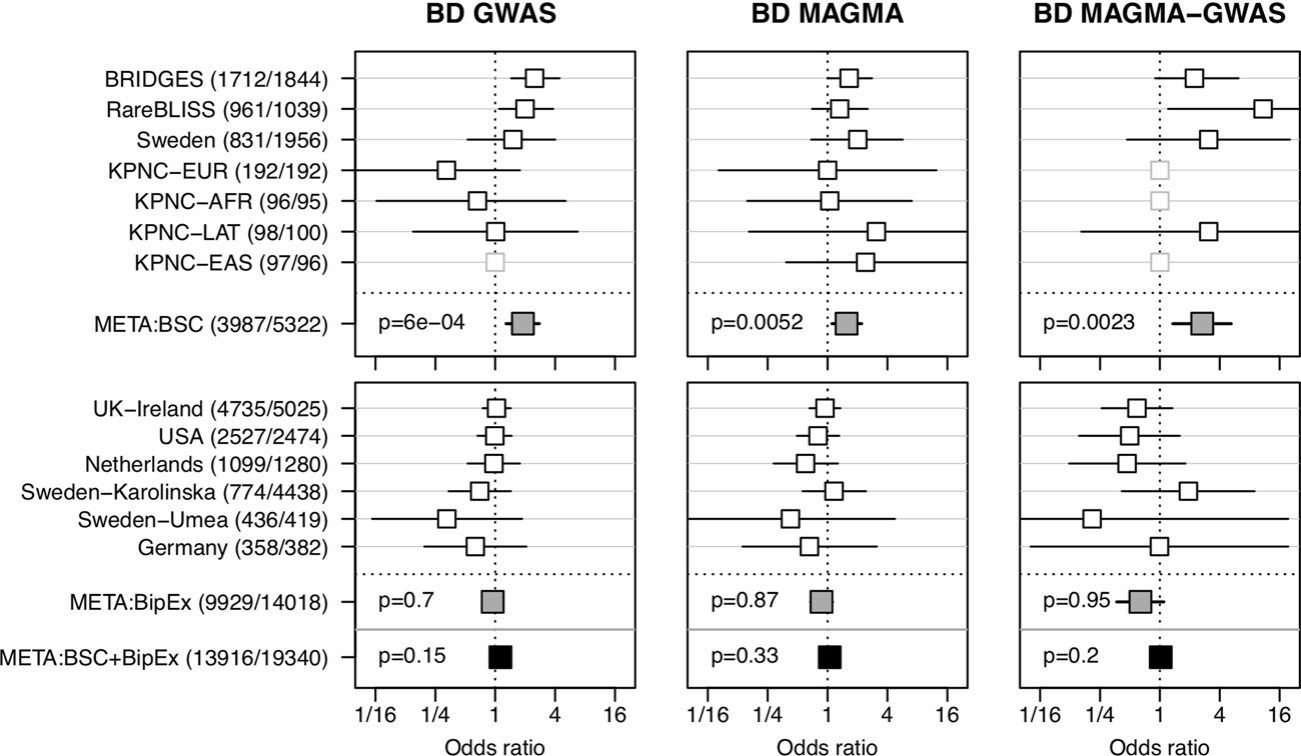
P-LP variant burden in BD GWAS gene sets within BSC discovery and BipEx replication cohorts. Forest plots of log odds ratios (Firth logistic regressions) for association between P-LP variant burden and BD across seven cohorts/ethnicities in the BSC study (top) and across six strata in the BipEx replication cohort (bottom). *p* values are one-sided for enrichment of P-PL variants in BD cases and derived from the meta-analysis Z-score. Meta-analysis shows that individuals with BD do not carry a replicable enrichment of P-LP variants in 165 BD GWAS-derived genes, in 153 BD genes identified using MAGMA, or in 81 genes that overlap between the BD GWAS and BD MAGMA gene sets. Horizontal black lines indicate 95% confidence intervals around the effect size. Unshaded boxes indicate absence of P-LP variants within a specific cohort. Gray boxes indicate meta-analysis within the BSC (discovery) and BipEx (replication) cohorts, respectively, and black box indicates meta-analysis across all BSC and BipEx cohorts. Numbers in parentheses indicate the number of BD cases and controls. BSC Bipolar Sequencing Consortium, BipEx Bipolar Exomes collection, REP replication cohorts, META meta-analysis.

However, BD cases carried a higher burden of P-LP variants compared to controls within 165 bipolar GWAS genes (meta-analysis of Firth logistic regressions OR = 1.89, 95% CI = 1.29–2.77, one-sided *p* = 6.0 × 10^−4^) and within 153 bipolar genes identified using MAGMA (meta-analysis OR = 1.56, 95% CI = 1.11–2.19, one-sided *p* = 0.0052) (Figs. 2, 3, Tables 2, S4 and S5). The relative burden of rare P-LP variants was higher within 81 genes representing the intersection of these two gene sets (meta-analysis OR = 2.66, 95% CI = 1.35–5.21, one-sided *p* = 0.0023) (Figs. 2, 3, Tables 2, S4 and S5). The CMH analysis results were consistent with those from meta-analysis of Firth logistic regressions described above and there was no evidence of heterogeneity in OR among the studies (Tables S4 and S5 for individual BSC study results). Meta-analysis results were also broadly consistent when subset to subjects of European ancestry (Tables S4 and S5).

**Table 2:** Rare variant burden in candidate gene sets.

Gene sets examined					Meta-analysis of Firth logistic regressions		
Gene set	Genes		P-LP count		OR (95% CI)	One-sided *p* value	Adjusted *p* value
	All	Carrying P-LP variants	3987 cases	5322 controls			
BD GWAS	165	35	78	51	1.89 (1.29–2.77)	0.0006	0.006
BD MAGMA	153	29	72	53	1.56 (1.11–2.19)	0.0052	0.042
BD MAGMA-GWAS	81	15	21	8	2.66 (1.35–5.21)	0.0023	0.021
SCZ GWAS	623	111	288	299	1.19 (1.02–1.39)	0.011	0.077
SCZ MAGMA	550	113	282	331	1.07 (0.89–1.28)	0.24	1.00
SCZ MAGMA-GWAS	342	72	198	219	1.11 (0.94–1.32)	0.12	0.72
Synaptic-RBFOX2	3,055	698	1,219	1,555	0.99 (0.98–1.01)	0.86	1.00
Synaptic-FMRP	1,233	413	756	976	0.92 (0.83–1.04)	0.91	1.00
LOF-intolerant	3,230	1,246	1628	2,059	0.93 (0.86–1.02)	0.94	1.00
All genes	25,134	2,634	7,847	9,787	1.00 (0.97–1.03)	0.39	1.00

For each of 10 candidate gene sets, we performed a meta-analysis of the effect of P-LP variant burden on BD within each study (using adjusted Firth logistic regression), and calculated a one-sided *p* value examining enrichment of P-LP variants in BD cases. Adjusted *p* values represent correction for multiple comparisons using the Holm method.

*BD* bipolar disorder, *SCZ* schizophrenia, *GWAS* genome-wide association study, *LOF* loss-of-function, *OR* odds ratio.

Attempted replication in WES data from 9,929 BD cases and 14,018 controls (Table S6) from the BipEx collection did not reproduce the observed enrichment of rare P-LP variants within the BD GWAS (meta-analysis OR = 0.94, one-sided *p* = 0.70), BD MAGMA (OR = 0.87, *p* = 0.87), or BD MAGMA-GWAS (OR = 0.64, one-sided *p* = 0.95) gene sets (Fig. 3, Table S7). Similarly, examination of 2,684 B1D-only cases and 14,018 controls did not reveal an enrichment of rare P-LP variants in the BD GWAS (OR = 0.89, one-sided *p* = 0.74), BD MAGMA (OR = 0.98, one-sided *p* = 0.54), or BD MAGMA-GWAS (OR = 0.85, one-sided *p* = 0.65) gene sets (Table S7). Thus, the initial observed enrichment of rare functional protein-altering variants within BD GWAS genes was not replicated.

Given the shared clinical features and reports of shared common genetic susceptibility between BD and SCZ, we also examined the burden of rare P-LP variants for three schizophrenia-related gene sets. Meta-analysis across cohorts showed no significant enrichment of rare P-LP variants in the 623 SCZ GWAS genes (OR = 1.19, 95% CI = 1.02–1.39, one-sided *p* = 0.01, adjusted one-sided *p* = 0.08), in 550 SCZ MAGMA genes (OR = 1.07, 95% CI = 0.89–1.28, one-sided *p* = 0.24), and in 342 SCZ MAGMA-GWAS genes (OR = 1.11, 95% CI = 0.94–1.32, one-sided *p* = 0.12) (Fig. 2, Tables 2, S4 and S5). CMH analysis again showed results similar to meta-analysis with Firth logistic regression (Tables 2 and S4). This suggests that despite the shared common genetic susceptibility between BD and SCZ, we found no evidence for BD risk being associated with rare functional protein-altering variation in schizophrenia GWAS genes.

We examined three gene sets previously reported to harbor rare variants that influence neuropsychiatric traits: 3,055 genes whose mRNAs are bound by the synaptic protein RBFOX2, 1,233 FMRP-related genes that co-localize with neuronal synapses, and 3,230 genes reported to be intolerant to loss-of-function mutations. Each of these gene sets has been implicated in rare variant studies of schizophrenia [26, 32, 34, 50, 52]. We observed no significant enrichment of rare P-LP variants in the RBFOX2-related genes (meta-analysis OR = 0.99, 95% CI = 0.98–1.01, one-sided *p* = 0.86), FMRP-related genes (OR = 0.93, 95% CI = 0.82–1.04, one-sided *p* = 0.91), or LOF-intolerant genes (OR = 0.94, 95% CI = 0.86–1.02, one-sided *p* = 0.94) in BD cases versus controls across studies (Fig. 3). CMH analysis confirmed a lack of rare variant enrichment within these gene sets (Table 2), thus we had no evidence of BD risk from rare functional protein-altering variants in a broad set of genes encoding proteins that localize to neuronal synapses, that were previously associated with schizophrenia.

Lastly, we used published PGC2 case-control GWAS meta-analysis results for BD and schizophrenia to test the common polygenic burden on risk of BD in our BSC subjects. Consistent with previous publications [60], common variant PRS for both BD and schizophrenia were highly significant in the combined meta-analysis (p_BD_ = 1.52 × 10^−41^ and p_SZ_ = 1.51 × 10^−32^, Table S8), but explained only a small proportions of variance in disease risk (~1–4%) in individual BSC studies, again demonstrating the shared genetic etiology of these psychiatric conditions, at least from common variants and in contrast to rare variant analyses.

## Discussion

To our knowledge, this primary analysis of 3,987 BD cases and 5,322 controls, along with independent replication/second stage samples of 9,929 cases and 14,018 controls represents the largest study of coding sequence variation in BD. We examined the exomes of these individuals, and identified rare coding variants classified as P-LP according to ACMG criteria [42, 44]. We did not observe a significant enrichment of rare P-LP variants in BSC BD cases versus controls overall or for any individual gene. We also did not observe an increased burden of rare P-LP variants in genes implicated by BD GWAS regions in the combined BSC and BipEx cases. This observation diverges from studies in some other complex disorders that found an enrichment of rare coding variation in genes associated with GWAS loci identified from common variants and vice versa [19–21, 61].

We did not observe a difference in rare P-LP variant burden within gene sets that influence synaptic function (RBFOX2 and FMRP), genes that are intolerant to loss-of-function mutations, or all genes across the exome. This diverges from prior studies of exonic variation in familial BD cases that suggest a role for synaptic-related genes [25], ion channels [23], and modulation of neurotransmitter activity [24]. However, those studies were small (far smaller than ours) and their findings unreplicated, highlighting the need for large studies with replication cohorts. While this study was not powered to identify novel single gene associations, it provides important insight into the genetic architecture of rare coding variants in BD, specifically the absence of replicable enrichment of such variants in several biologically plausible gene sets.

A shared genetic susceptibility to BD and SCZ is suggested through investigations of genome-wide common variation and polygenic risk scores (PRS) [12, 27–29]. Our results confirmed the correlation of common SCZ variants with BD risk. Moreover, examination of sub-phenotypes suggests that the SCZ PRS is associated with psychosis in BD, and that the BD PRS is associated with mania in SCZ [23, 24]. However, examination of rare coding variation does not support a clear role for BD-related genes in SCZ risk [32]. Similarly, we observe no significant enrichment of rare P-LP variants within SCZ GWAS genes in individuals with BD, or in other gene sets (RBFOX2, FMRP, LOF-intolerant, exome-wide) that have been reported to harbor rare variants in SCZ [32, 35]. While larger studies might reveal a smaller effect within these broader gene sets, this study does not show a clear role for rare variants within SCZ-related genes in BD.

The absence of deleterious rare variant enrichment in BD cases contrasts to studies of SCZ of similar size (4–5 K cases, 7–9 K controls) which show that individuals with SCZ carry an increased burden of rare protein-truncating variants across the exome, particularly in genes intolerant to loss-of-function mutations [32, 35]. This burden was particularly elevated in SCZ cases with intellectual disability [35]. Another study showed that rare CNVs are enriched in individuals with schizoaffective disorder bipolar type (SAB), but not in all individuals with BD [62]. These observations suggest that, while there exists a shared susceptibility for BD and SCZ through common genetic variation, rare coding variation in gene sets implicated in SCZ might preferentially confer a risk for SCZ-specific features independent of the affective symptoms that are definitional of BD. Thus, individuals with rare variants in genes implicated in SCZ might present with psychotic or cognitive predominant symptoms, rather than manic or hypomanic episodes, which are required for a diagnosis of BD.

In this study, variants classified as pathogenic are loss-of-function (LOF) mutations (splice-site and stop-gain) in a gene where LOF is a known mechanism of disease, while those classified as likely pathogenic are primarily missense variants in a gene with a low rate of benign missense variation. Both types of variants are absent or at very low frequency in large control cohorts (gnomAD), and have multiple lines of evidence for a deleterious effect. While there are likely a number of P-LP variants not included as P-LP in our study (e.g., frameshift LOF variants), their non-inclusion would only have limited the power of our analysis, and not created a bias. Nonetheless, examination of the P-LP variants we included did enable systematic interrogation of candidate gene sets that might be biologically relevant for a complex phenotype such as BD.

Although we found three significantly associated genesets in our BSC study, these genesets did not replicate in the BipEx study and they were not significant in a meta-analysis of the BSC and BipEx studies. There are differences in the case composition of the BSC and BipEx samples. More BD cases were classified as B1D, a form of BD with more extreme episodes of mood elevation [63], in the BSC discovery cohort (91% of cases) than in the BipEx replication cohort (74% of cases with known subtypes), However, when restricting to the smaller set of BipEx B1D cases we still did not detect association in the genesets identified in the BSC studies. It may be that the BSC geneset-based results are false positives or that larger sample sizes with deleterious variants defined by other metrics may be necessary to consistently detect a signal.

We acknowledge a number of limitations. First, there are a number of inter-study differences in methods used for case-control selection, DNA sample acquisition, exome or genome sequencing, and data quality control. While these differences might contribute to biases in variant detection in cases and controls, we showed that our approach produced no exome-wide deviation of rare synonymous variants in cases versus controls. Second, our analysis was limited to coding single nucleotide variants, which have the highest quality annotation in large public databases (gnomAD) and annotation tools (InterVar). Future WGS studies that include analysis of copy number, insertion-deletion, and regulatory variants may shed further light on the role of rare variants in BD. Third, while genome-wide common variant studies implicate loci that influence susceptibility for BD, many genes in these regions that were included in our analysis are likely unrelated to BD, highlighting the need for fine-mapping and functional studies. Lastly, our ability to further investigate the observed differences between the discovery and replication cohorts was limited due to the lack of deep sub-phenotype data (such as severity of mood episodes, psychosis, hospitalizations) in the majority of BD cases.

Despite the limitations, this study provides important biologic insight into disease pathogenesis in BD. While common and rare variant associations implicate several genes critical for neuronal development and synaptic function [15–18, 22–26], this study suggests that BD risk may not be broadly influenced by rare coding variation in genes within these categories. Moreover, while common genetic association studies suggest a role for shared genetic susceptibility between BD and SCZ, there is limited evidence for a shared susceptibility from rare genetic variation for these related mental disorders. Additional studies are needed to dissect the relationship between rare coding variants and subtypes of BD, including SAB. Neuroimaging and neurophysiological biomarkers [64, 65] may be used to identify genetic factors that influence endophenotypes within these complex disorders. Lastly, loci identified through BD common variant studies likely require fine-mapping through functional studies, and not only through scalable genomics methods, for translational impact.

## Supplementary information

Supplementary Materials

Supplementary Tables
