# Supplementary material for: Investigating rare pathogenic/likely pathogenic exonic variation in bipolar disorder
Source: Mol Psychiatry. 2021 Jan 22;26(9):5239–50. doi: 10.1038/s41380-020-01006-9 (PMC8295400; doi:10.1038/s41380-020-01006-9)
Supplement: Supplementary file 1 — Supplementary Materials [file 41380_2020_1006_MOESM1_ESM.docx]

**SUPPLEMENTAL METHODS**

**Study cohorts**

All human studies were approved by each respective institutional ethics review committee, and all participants provided written informed consent. To investigate the role of rare coding variation in the pathogenesis of bipolar disorder (BD), we examined the protein-coding sequences (exomes) of 3,987 individuals with BD and 5,322 healthy individuals from four case-control cohorts of predominantly European ancestry in the Bipolar Sequencing Consortium (Table 1 and Fig. 1, Fig S1, Fig S2).

BRIDGES cohort

The BRIDGES study included 3,556 individuals of European descent from six different cohorts in the US, Canada, and UK: Prechter Repository ^1^, STEP-BD cases from the NIMH repository ^2^, the Genomic Psychiatry Cohort ^3^, the Minnesota Center for Twin and Family Research (MCTFR) ^4^, Centre for Addiction and Mental Health (CAMH) in Toronto, and the Institute for Psychiatry, Psychology and Neuroscience (IOPPN) at King’s College London ^5^. The STEP-BD samples consisted only of cases from the NIMH repository. MCTFR samples consisted of only control individuals matched to STEP-BD cases and unmatched cases from the Prechter repository. Cases from all studies had psychiatrist confirmed diagnoses with bipolar 1 disorder (B1D) with or without psychosis while controls had no history of psychiatric illness.

RAREBLISS cohort

The RareBliss sample originally consisted of 1,135 BD cases and 1,142 unrelated controls.  As the preliminary pilot samples were sequenced on early exon target capture kits, we restricted our analyses to 961 cases 1,039 and controls that were sequenced using a uniform Nimblegen SeqCap EZ Exome v2 capture kit.  All cases and controls were obtained from the NIMH Genetics Initiative repository (https://nimhgenetics.org/) and were of self-identified European-American descent. Cases were originally ascertained as part of the National Institute of Mental Health (NIMH) Genetics Initiative BD Collaborative study waves 1-5. All subjects were assessed with the Diagnostic Interview for Genetic Studies (DIGS) ^6^ followed by a best-estimate diagnostic procedure according to DSM-III-R, or DSM-IV criteria. Among the 961 cases included in this study, 873 (89.8%) were diagnosed with bipolar I disorder (BPI) and 88 (10.2%) were diagnosed with schizoaffective disorder, bipolar type (SAB). The 1,069 controls were obtained from the NIMH Genetics Initiative repository and were originally recruited by Knowledge Networks, Inc. (Menlo Park, CA) from a nationally representative marketing panel ^7^.  All control subjects completed an online version of the Composite International Diagnostic Interview-Short Form (CIDI-SF), which assesses common mood, anxiety and substance use disorders ^8^.  The CIDI-SF was supplemented by questions about any history of schizophrenia, psychosis or BD. Completion of these supplemental questions was required for inclusion in the study.

SWEDEN cohort

The SWEDEN study consists of a total of 12,384 blood-derived DNA samples collected from 2005 to 2013, including 4,970 schizophrenia samples, 1,169 bipolar samples and 6,245 controls. Participants with a psychiatric diagnosis were ascertained from the Swedish National Hospital Discharge Register and controls were randomly selected from population registers. After quality control (see sequencing and quality control), a total of 831 bipolar cases and 1,956 matched controls were included in this study. Among the 831 cases included in this study, 547 (65.8%) were diagnosed with B1D, 252 (30.3%) with B2D, 3 (0.3%) with SAB, 14 (1.7%) with NOS and 15 (1.8%) with bipolar disorder subtype information missing.

KPNC cohort

The Kaiser Permanente Northern California (KPNC) cohort included 483 individuals with B1D from the Multi-ethnic Study of Bipolar Disorder (MSBP), and 483 ancestry-matched controls ^9^. All 483 BD cases included in this study had diagnoses of B1D; individuals diagnosed with schizoaffective disorder were excluded. Ethnicities represented in this cohort included European (EUR, 192 cases, 192 controls), African American (AFR, 96 cases, 95 controls), Latino (LAT, 98 cases, 100 controls), and East Asian (EAS, 97 cases, 96 controls). Healthy controls were matched to bipolar cases according to genotype-determined ancestry from four ancestry-specific genotyping arrays ^10-12^.

**Replication cohorts**

To replicate any positive findings from the BSC cohort, we examined the exomes of 9,929 individuals with BD and 14,018 healthy individuals from six cohorts of European ancestry from the Bipolar Exomes (BipEx) collection. Of 3,639 BD cases with known subphenotypes, 2,684 were classified as B1D, and 955 were classified as B2D. Individuals with schizophrenia and schizoaffective disorder recruited through this Initiative were excluded in this analysis. The six cohorts within the Dalio Initiative included subjects from the UK and Ireland (4,735 BD cases and 5,025 controls), US (2,527 BD cases and 2,474 controls), Netherlands (1,099 BD cases and 1,280 controls), Sweden-Karolinska (774 BD cases and 4,438 controls), Sweden-Umea (436 BD cases and 419 controls), and Germany (358 BD cases and 382 controls).

Samples from UK and Ireland were obtained from the University of Aberdeen (332 controls), Trinity College Dublin (203 BD cases and 634 controls), University of Edinburgh (936 BD cases and 37 controls), University College London (2,228 BD cases and 1,330 controls), Cardiff University (2,441 BD cases and 1,112 controls), and University of Cambridge (2,873 controls). Samples from the United States were obtained from the Massachusetts General Hospital and Johns Hopkins University (3,895 BD cases and 3,627 controls). Samples from the Netherlands were obtained from the University of California, Los Angeles (2,280 BD cases and 636 controls of Dutch ancestry) and Vrije Universiteit Amsterdam (951 controls). Samples from Karolinska (Sweden) were obtained from the Karolinska Institutet (5,175 BD cases obtained from the HD registry, QA registry and St. Goran Hospital) and LifeGene Biorepository within Karolinska (5,560 controls). Samples from Umea (Sweden) were obtained from Umea University (488 BD cases and 488 controls). Samples from Germany were obtained from Universitätsklinikum Frankfurt (414 BD cases and 416 controls).

**Sequencing and quality control**

BRIDGES methods

Full details of the BRIDGES sequencing study have been published previously ^13^. In brief, whole genome sequencing was performed for each individual on the Illumina HiSeq 2500 using 100bp paired-end reads. Individually barcoded samples were sequenced in pools of 12 to an average coverage of 9.6 per sample and cases and controls were evenly included in the pools. Sequencing reads were aligned to build hg19 of the human reference and single nucleotide variants were called using the GotCloud sequence analysis pipeline according to standard recommendations ^14^. Samples that failed sequencing, were improperly paired read, had sequence coverage < 3x, had estimated contamination >3%, and had unresolved sample swaps were excluded. Following variant calling, principal component-based population outliers, samples showing systematic sequencing artifacts, and samples with cryptic relatedness were also removed. After removing problematic samples, further variant level QC as recommended in GotCloud was performed and variants with a Hardy-Weinberg equilibrium P-value < 1E-6 were removed.

RAREBLISS methods

Exome sequencing in 961 BD cases and 1,039 controls was performed using the Nimblegen SeqCap EZ Exome v2 capture kit, and included custom targets of regulatory regions from 1,422 genes that encode proteins in neuronal synapses and 60 genes previously implicated in BD. Samples were sequenced using Illumina HiSeq 2000, and were included in this study if at least 70% of the target sequence was covered at 20X or more. Alignment was performed using BWA ^15^, followed by indexing, and quality control via SAMtools ^16^ and BAMtools ^17^. Picard ([http://picard.sourceforge.net](http://picard.sourceforge.net/)) was used to fix mismatched mate pairs and to remove PCR duplicates. Cases and controls were included in each of the three rounds of sequencing. Target coverage was assessed using Picard’s HSmetrics utility. The Genome Analysis Toolkit (GATK) ^18^ was used for indel realignment followed by joint variant calling using the Unified Genotype caller. SNVs in regions that might represent false-positives were removed, including SNVs in a region with greater than three SNVs per ten bases, and SNVs falling within putative indels.  Any genotypes with read depth < 10 and genotype quality score < 20 were also removed, as well as SNVs with > 10% missing calls or in Hardy-Weinberg Disequilibrium (P < 1x10^-6^).

SWEDEN methods

Sequencing was performed at the Broad Institute between 2010 and 2013. A total of 12,384 samples were sequenced in 12 separate waves, with bipolar cases in wave 8, 9 and 10 without controls. Hybrid-capture using the Agilent SureSelect Human All Exon v.2 Kit was performed, along with exome sequencing using Illumina HiSeq 2000 and HiSeq 2500 instruments, with paired-end sequencing reads of 76 base pairs. Sequence alignment to human genome reference GRCh37 was performed using bwa v0.5.9, and variant calling was performed using the GATK Haplotype Caller version 3.1. Calls that were filtered by the GATK Variant Quality Score Recalibration (VQSR) tool, and which had less than 10x coverage, were excluded. After variant calling, principal component-based population outliers, sample duplicates and relatives, and samples showing systematic sequencing artifacts were removed. Further individual-level quality control in 1,169 bipolar cases and 6,245 controls was performed by keeping individuals that met all of the criteria: (1) birth year between 1928 and 1985, (2) mean target coverage > 50X, and (3) meet additional metrics of exome-wide variant quality. Bipolar cases were matched with controls using Mahalanobis distance based on the number of non-reference variants per sample and sequence quality metrics, and a final cohort including 831 bipolar cases and 1,956 matched controls was created. Finally, variant-level quality control was performed by removing SNVs that met one or more of the following criteria: (1) genotype quality < 20, (2) missingness > 0.1, and (3) Hardy-Weinberg equilibrium p-value < 10^-3^.

KPNC methods

Whole-exome sequencing in 966 individuals from KPNC was performed using the Illumina HiSeq 2500 system. DNA library capture was performed using the NimbleGen SeqCap EZ Exome V3 system. Cases and controls were sequenced together. Samples were sequenced at 30X coverage, 100 bp paired DNA reads were aligned to the human genome (reference hg19, build 37) using bwa ^15^, duplicate reads were removed using samtools, local realignment and base recalibration was performed using Picard and Genome Analysis Toolkit (GATK) ^18^, and joint calling of single nucleotide variants (SNVs) was performed using the GATK UnifiedGenotyper V2.6-4. Low-confidence SNV calls were filtered out by removing variants with less than 10X coverage and those outside of known exome capture target regions. Variant-level quality control for individuals in KPNC was performed by removing SNVs that met one or more of the following criteria using Plink ^19,20^: (1) missingness > 0.2, (2) Hardy-Weinberg equilibrium p-value < 10^-6^, and (3) differential missingness in cases and controls p-value < 0.001.

Replication cohort methods

Whole exome sequencing was performed in 13,207 bipolar cases and 16,964 controls that comprised the BipEx collection. Samples were processed as they were received in cohorts from collaborators. Sample QC was performed using the picogreen assay to measure sample volume, concentration and DNA yield. Sample library was prepared using Illumina Nextera, followed by hybrid capture using Illumina rapid capture enrichment of 37Mb target. Sequencing was performed on HiSeqX instruments to 150bp paired reads. This process typically yields ~55x mean coverage. Each sample’s sequencing reads were aggregated into a BAM file and processed through a pipeline based on the Picard set of software tools. The BWA aligner was used to map reads onto the human genome build 37 (hg19). Single nucleotide polymorphism and insertions/deletions were jointly called across all samples using GATK HaplotypeCaller package version 3.4 to produce a version 4.1 variant call set file (VCF). Variant call accuracy was estimated using the GATK Variant Quality Score Recalibration (VQSR) approach.

After variant calling, low-confidence calls (genotype quality < 20, or fraction of reads consistent with call < 80%) and those with coverage < 10x were removed. Variants that were outside of the exome target interval and those failing additional quality control measures (failed variant quality score recalibration, call-rate < 94%, mean depth < 30, mean genotype quality < 85, percentage contamination > 0.2%, percentage of chimeric reads > 0.15%) were removed. Individuals with inconsistencies in sex data and those found to be related (p̂ > 0.2 from identity by descent analysis) were removed. Principal component analysis (PCA) on sequenced samples and 1000 genomes samples was used to identify and keep individuals of European ancestry. Individuals of non-European or Ashkenazi Jewish ancestry were removed due to paucity of ancestry matched case-control samples. Finally, variants that had a call rate below 97% (across all samples, or in cases or controls), absolute difference in call rate between cases and controls greater than 2%, or HWE p-value < 1E-6 were removed. Individuals with sequencing metrics outside of three standard deviations from the mean (transition to transversion ratio, heterozygous to homozygous alternative allele ratio, insertion to deletion ratio) were removed. The resultant collection of samples and variants consisted of 9,929 bipolar cases and 14,018 ancestry matched controls, and a total of 4,351,347 variants.

**Gene set burden testing**

First, for each gene set, in each study (and within each race/ethnicity group in KPNC) and for cases and controls separately, we determined the total number of rare (gnomAD maximum population MAF < 1%), protein-altering (missense, splice site, stopgain, startloss), single nucleotide variants that were classified as pathogenic (P) or likely pathogenic (LP) using InterVar. We assessed whether individuals with BD carry a higher burden of P-LP variants in a set of candidate genes (in which disrupted protein function is the presumed mechanism for increased BD risk). We performed Firth logistic regression on the burden of P-LP alleles per individual within seven groups (BRIDGES, RAREBLISS, Sweden, KPNC-EUR, KPNC-AFR, KPNC-LAT, KPNC-EAS) followed by a meta-analysis across cohorts using the weighted Z-score method (weighted by the square root of total sample size of each cohort) ^21^. For each gene set, we summed the number of P-LP alternate alleles per individual, and performed Firth logistic regression (‘logistf’ package in R) on this allele count adjusted for several study-defined covariates (BRIDGES: sex plus top 10 ancestry principal components [PCs] derived from DNA sequence data; RAREBLISS: sex plus top 5 ancestry PCs, KPNC: sex plus top 5 ancestry PCs within each of four race/ethnicity groups; Sweden: sex, top 5 ancestry PCs, exome-wide burden of P-LP variants outside the gene set, and exome-wide burden of non-reference alleles). We then calculated the study-specific (one-sided) Z-scores using the adjusted Firth logistic regression p-values, and performed a meta-analysis across studies using the weighted Z-score method (weighted by the square root of total sample size of each cohort). Supplemental Figure 2 shows the first two principal components (PC) for each dataset. We determined the meta-analysis effect size using the inverse variance-weighted method, and calculated 95% confidence intervals using the resulting meta-analysis effect size and Z-score.

We next performed a Cochran-Mantel-Haenszel (CMH) chi-square test across studies. We determined the P-LP variant count by summing their number across individuals (count of 1 for heterozygous carrier and 2 for homozygote), and the non-carrier count by summing the number of reference alleles for the same variants. We used the CMH test to determine the odds ratio (OR) and 1-sided chi-square and p-value. We then assessed whether the variant burden differed across studies using a Breslow-Day test for heterogeneity.

We assessed statistical significance by a simulation analysis. For each gene set, we randomly selected an equivalent number of genes in 10,000 simulations, and generated a null distribution for the CMH chi-square statistic and odds ratio, based on the total number of P-LP variants in cases and controls found in the simulated gene set. We subsequently determined the empirical p-value for observing an enrichment of deleterious variants in BD cases by comparing the observed CMH chi-square statistic to the randomly generated null distribution. We performed these random simulations to control for potential confounding due to variability in sample selection and sequencing methods between cases and controls within each study.

**Supplemental References**

1 Langenecker, S. A., Saunders, E. F., Kade, A. M., Ransom, M. T. & McInnis, M. G. Intermediate: cognitive phenotypes in bipolar disorder. *Journal of affective disorders* **122**, 285-293, doi:10.1016/j.jad.2009.08.018 (2010).

2 Sklar, P. *et al.* Whole-genome association study of bipolar disorder. *Molecular psychiatry* **13**, 558-569, doi:10.1038/sj.mp.4002151 (2008).

3 Pato, M. T. *et al.* The genomic psychiatry cohort: partners in discovery. *American journal of medical genetics. Part B, Neuropsychiatric genetics : the official publication of the International Society of Psychiatric Genetics* **162b**, 306-312, doi:10.1002/ajmg.b.32160 (2013).

4 Miller, M. B. *et al.* The Minnesota Center for Twin and Family Research genome-wide association study. *Twin research and human genetics : the official journal of the International Society for Twin Studies* **15**, 767-774, doi:10.1017/thg.2012.62 (2012).

5 Scott, L. J. *et al.* Genome-wide association and meta-analysis of bipolar disorder in individuals of European ancestry. *Proc Natl Acad Sci U S A* **106**, 7501-7506, doi:10.1073/pnas.0813386106 (2009).

6 Nurnberger, J. I., Jr. *et al.* Diagnostic interview for genetic studies. Rationale, unique features, and training. NIMH Genetics Initiative. *Archives of general psychiatry* **51**, 849-859; discussion 863-844 (1994).

7 Sanders, A. R. *et al.* No significant association of 14 candidate genes with schizophrenia in a large European ancestry sample: implications for psychiatric genetics. *The American journal of psychiatry* **165**, 497-506, doi:10.1176/appi.ajp.2007.07101573 (2008).

8 Kessler, R. C. *et al.* Screening for serious mental illness in the general population. *Archives of general psychiatry* **60**, 184-189 (2003).

9 Banda, Y. *et al.* Characterizing Race/Ethnicity and Genetic Ancestry for 100,000 Subjects in the Genetic Epidemiology Research on Adult Health and Aging (GERA) Cohort. *Genetics* **200**, 1285-1295, doi:10.1534/genetics.115.178616 (2015).

10 Kvale, M. N. *et al.* Genotyping informatics and quality control for 100,000 subjects in the genetic epidemiology research on adult health and aging (GERA) cohort. *Genetics*, doi:10.1534/genetics.115.178905 (2015).

11 Hoffmann, T. J. *et al.* Next generation genome-wide association tool: Design and coverage of a high-throughput European-optimized SNP array. *Genomics*, doi:10.1016/j.ygeno.2011.04.005 (2011).

12 Hoffmann, T. J. *et al.* Design and coverage of high throughput genotyping arrays optimized for individuals of East Asian, African American, and Latino race/ethnicity using imputation and a novel hybrid SNP selection algorithm. *Genomics*, doi:10.1016/j.ygeno.2011.08.007 (2011).

13 Carlson, J. *et al.* Extremely rare variants reveal patterns of germline mutation rate heterogeneity in humans. *Nat Commun* **9**, 3753, doi:10.1038/s41467-018-05936-5 (2018).

14 Jun, G., Wing, M. K., Abecasis, G. R. & Kang, H. M. An efficient and scalable analysis framework for variant extraction and refinement from population-scale DNA sequence data. *Genome research* **25**, 918-925, doi:10.1101/gr.176552.114 (2015).

15 Li, H. & Durbin, R. Fast and accurate short read alignment with Burrows-Wheeler transform. *Bioinformatics* **25**, 1754-1760, doi:10.1093/bioinformatics/btp324 (2009).

16 Li, H. *et al.* The Sequence Alignment/Map format and SAMtools. *Bioinformatics* **25**, 2078-2079, doi:10.1093/bioinformatics/btp352 (2009).

17 Barnett, D. W., Garrison, E. K., Quinlan, A. R., Stromberg, M. P. & Marth, G. T. BamTools: a C++ API and toolkit for analyzing and managing BAM files. *Bioinformatics* **27**, 1691-1692, doi:10.1093/bioinformatics/btr174 (2011).

18 McKenna, A. *et al.* The genome analysis toolkit: A MapReduce framework for analyzing next-generation DNA sequencing data. *Genome research* **20**, 1297-1303, doi:10.1101/gr.107524.110 (2010).

19 Chang, C. C. *et al.* Second-generation PLINK: Rising to the challenge of larger and richer datasets. *GigaScience*, doi:10.1186/s13742-015-0047-8 (2015).

20 Purcell, S. *et al.* PLINK: a tool set for whole-genome association and population-based linkage analyses. *American journal of human genetics* **81**, 559-575, doi:10.1086/519795 (2007).

21. Liptak, T. 1958. On the combination of independent tests. Magyar Tud Akad Mat Kutato Int Kozl. 1958;3:171-96.

**
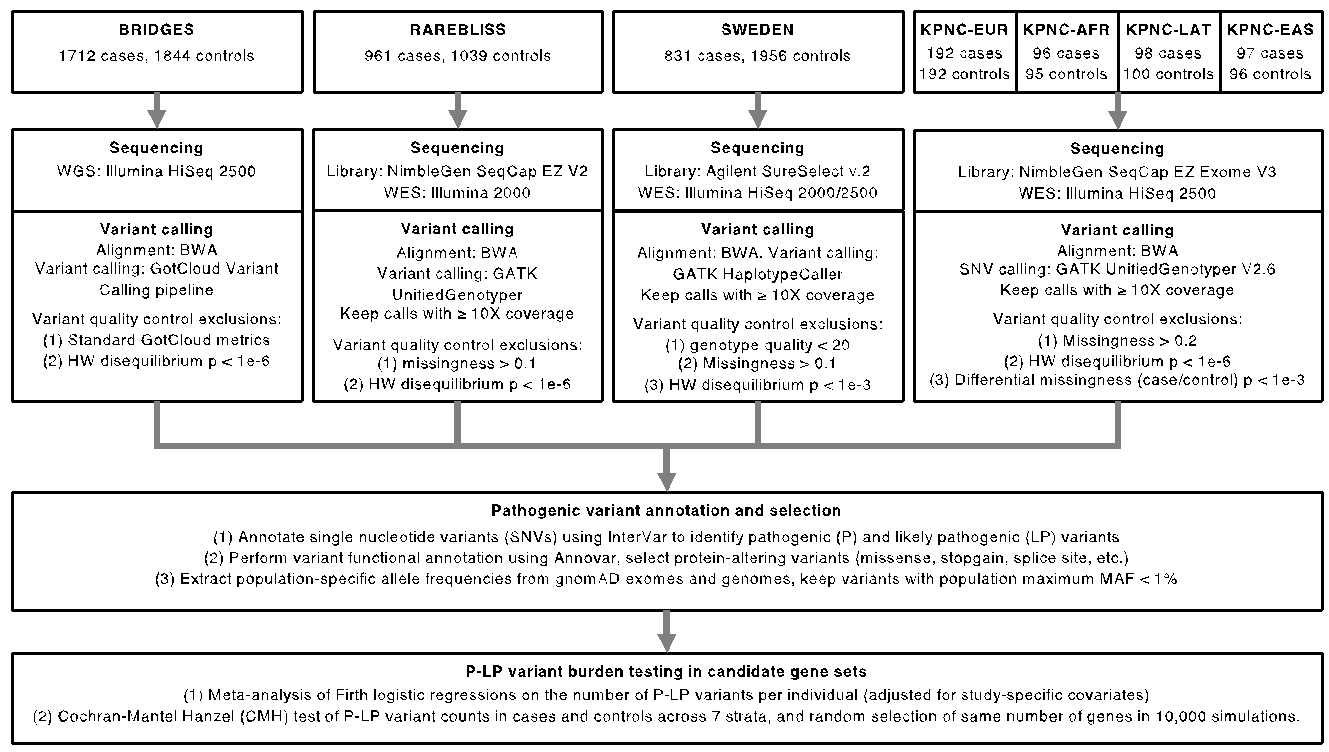
SUPPLEMENTAL FIGURES**

**Figure S1: Cohorts and methods used in bipolar sequencing consortium study.** A total of 3987 bipolar cases and 5322 controls of predominantly European ancestry received whole-exome (or whole-genome) sequencing across four studies. Variant calling and quality control were performed separately within each study to account for inter-study differences in sequence data acquisition. All variants were annotated to identify rare coding SNVs classified as pathogenic or likely-pathogenic according to ACMG criteria. Two statistical methods (meta-analysis of Firth logistic regressions, CMH test and random gene selections) were used to examine whether BD cases are enriched for P-LP variants within a number of pre-defined candidate gene sets.

BRIDGES: Bipolar Research in Deep Genome and Epigenome Sequencing Study. RareBLISS: Rare Bipolar Loci Identification through Synaptome Sequencing. KPNC: Kaiser Permanente Northern California. EUR: European ancestry. AFR: African American ancestry. LAT: Latino ancestry. EAS: East Asian ancestry. WGS: whole-genome sequencing. WES: whole-exome sequencing. GATK: Genome Analysis ToolKit. P-LP: Pathogenic and likely-pathogenic.

**
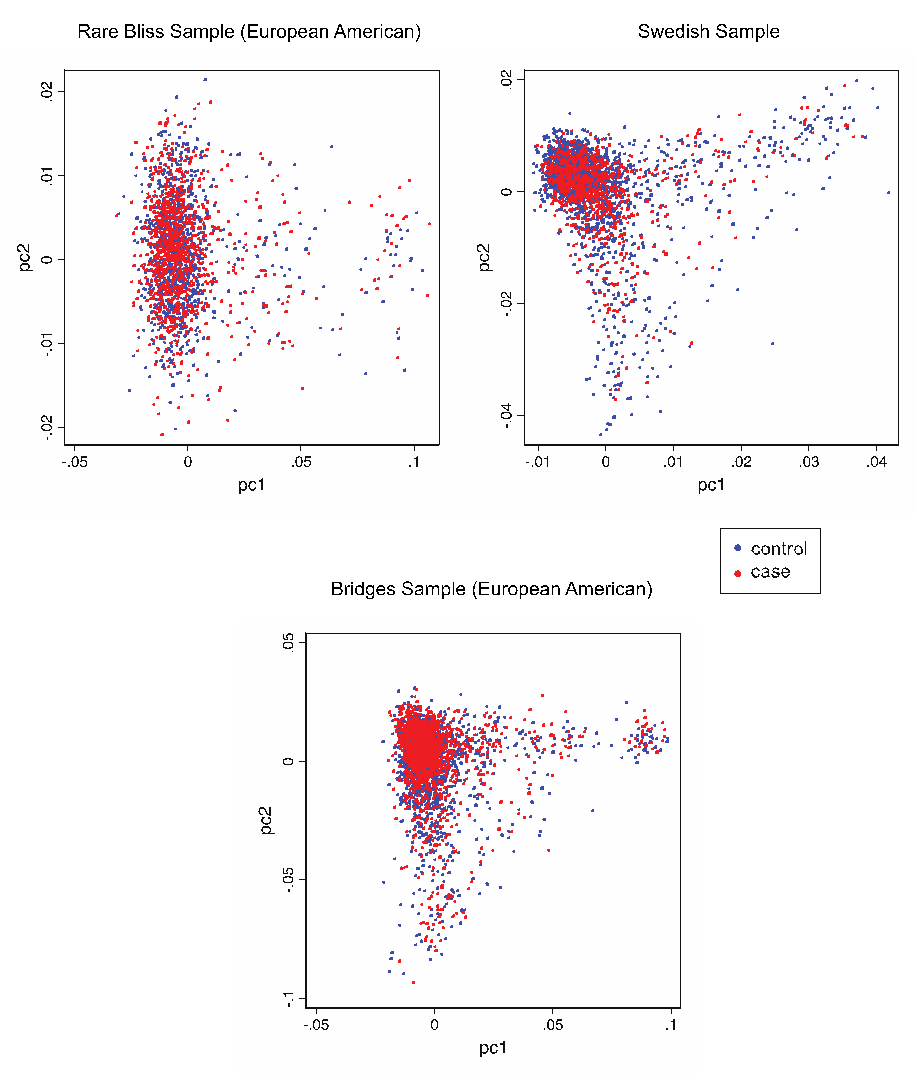

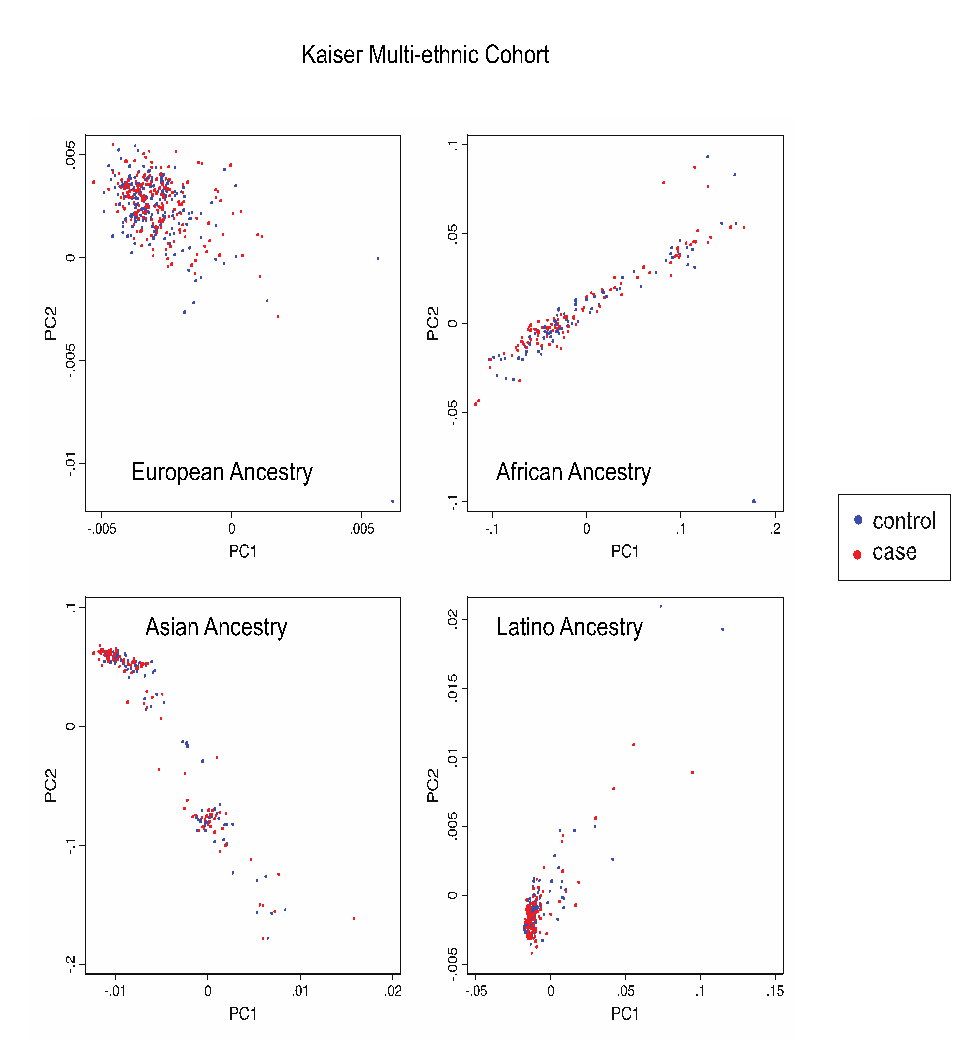
**

**Figure S2: Genetic ancestry distribution using principal components in the BSC cohorts**. Bipolar cases (red) and controls (blue) are plotted along the two axes of genetic variation (first two principal components) derived using principal component analysis (PCA) of coding sequence variation within each cohort/ethnicity (RareBliss, Sweden, BRIDGES, KPNC-EUR, KPNC-AFR, KPNC-LAT, and KPNC-EAS).
